# COVID-19 and instability of stock market performance: evidence from the U.S.

**DOI:** 10.1186/s40854-021-00229-1

**Published:** 2021-02-24

**Authors:** Hui Hong, Zhicun Bian, Chien-Chiang Lee

**Affiliations:** 1 Research Center for Central China Economic and Social Development, Nanchang University, Nanchang, Jiangxi China; 2 School of Economics and Management, Nanchang University, Nanchang, Jiangxi China; 3 School of Finance, Nanjing University of Finance and Economics, Nanjing, Jiangsu China

**Keywords:** COVID-19, Stock returns, Structural breaks, U.S, C22, G15, G18

## Abstract

The effect of COVID-19 on stock market performance has important implications for both financial theory and practice. This paper examines the relationship between COVID-19 and the instability of both stock return predictability and price volatility in the U.S over the period January 1st, 2019 to June 30th, 2020 by using the methodologies of Bai and Perron (Econometrica 66:47–78, 1998. 10.2307/2998540; J Appl Econo 18:1–22, 2003. 10.1002/jae.659), Elliot and Muller (Optimal testing general breaking processes in linear time series models. University of California at San Diego Economic Working Paper, 2004), and Xu (J Econ 173:126–142, 2013. 10.1016/j.jeconom.2012.11.001). The results highlight a single break in return predictability and price volatility of both S&P 500 and DJIA. The timing of the break is consistent with the COVID-19 outbreak, or more specifically the stock selling-offs by the U.S. senate committee members before COVID-19 crashed the market. Furthermore, return predictability and price volatility significantly increased following the derived break. The findings suggest that the pandemic crisis was associated with market inefficiency, creating profitable opportunities for traders and speculators. Furthermore, it also induced income and wealth inequality between market participants with plenty of liquidity at hand and those short of funds.

## Introduction

The novel coronavirus 2019 (COVID-19) disease has led to an unprecedented disruption to the U.S. economy and also an unparalleled slump in the U.S. stock market. Typically, a key market-wide circuit breaker designed to prevent the stock market from falling through the floor had been triggered four times in sequence in March 2020. Investors inevitably suffered heavy losses from plunging stock prices. Fears about the crisis and its impact on the global economy rapidly spread to the rest of the world. According to the report ‘Coronavirus: US stocks see worst fall since 1987 from China Daily on the 17th March 2020, after the U.S. market experienced worst point declines in history, global markets saw similar slides. That is, the performance of the U.S. market was a leading indicator of ups and downs of global markets particularly under such circumstances. This paper thus provides a comprehensive analysis on the association between COVID-19 and the instability of the U.S. stock market performance (including both return predictability and price volatility), which serves the interests of investors when making investment decisions during difficult times (Kou et al. 2014, 2021). Facts in the past prove that crises induce risks but also create investment opportunities: stock return predictability and price volatility substantially increase in bad times (e.g., Schwert 1989; Cujean and Hasler 2017; Hong et al. 2018; Liu et al. 2020a; Narayan 2020a, 2020b).

Since the outbreak of COVID-19, there are an increasing number of studies on stock market reactions to the crisis (e.g., Ashraf 2020a, b; Baker et al. 2020; Lee and Chen 2020; Mazur et al. 2020; Narayan et al. 2020; Topcu and Gulal 2020). The arrived consensus is that stock prices severely dropped and price fluctuation greatly enlarged following the occurrence of the pandemic disease. However, those studies did not test whether and when COVID-19 triggered the dramatic changes in stock market performance assuming no prior knowledge of the break location. Furthermore, they only focused on price changes and volatility but failed to concern return predictability which is an important theme in the finance literature.

This paper fills the research gap by studying the instability of stock return predictability and price volatility, and its linkage with COVID-19. Using daily data from January 1st, 2019 to June 30th, 2020 and the structural break tests proposed by Bai and Perron (1998, 2003), Elliot and Mullier (2004) and Xu (2013), we find that there exists a single break for both S&P 500 and DJIA return prediction models. The break took place in mid-late February, 2020, the timing of which is consistent with the COVID-19 outbreak or more specifically the stock selling-offs by the U.S. senate committee members before COVID-19 crashed the market. Furthermore, we also find evidence of a single break in price volatilities of both S&P 500 and DJIA, which occurred on February 21st, 2020, similar to the case of return predictability. The findings of significant increases in both return predictability and price volatility during COVID-19 indicate that the pandemic created profitable investment opportunities for market participants, with those with plenty of liquidity at hand benefiting most. Fed policy of pumping liquidity to the financial system might have stimulated profitability seeking in the stock market, which may enlarge income and wealth inequality.

The paper thus contributes to the existing literature in two important ways. First, previous studies either use event analyses (i.e., partition the sample to examine the difference of stock market performance across subsamples) or exogenous variable analyses (i.e., take major events as exogenous variables and establish models describing the relationship between market performance and those variables) to determine how stock markets react to external shocks (e.g., Mohanty et al. 2010; Ashraf 2020a, b). This paper tests for structural breaks in return predictive and price volatility models without COVID-19 variables and investigates the linkage between those breaks and COVID-19, thus providing explicit empirical evidence about whether and how public crisis affects market performance. Second, it also adds to the literature by studying stock return predictability when COVID-19 broke out, thus providing unprecedented evidence that suggests potential investment opportunities for investors.

The rest of the paper proceeds as follows. First, we briefly review the literature. Second, we describe the data used for empirical analysis. Third, we evaluate the role of COVID-19 on the instability of return predictability and price volatility in the U.S. market, respectively. Finally, we conclude the paper with suggestions to both investors and policy makers.

## Literature review

### Determination of future stock return and volatility

As for prediction, a large literature has identified a number of predictors that are useful to predict future stock returns. Those include (but are not limited to) dividend yield and dividend-price ratio (Fama and French 1988; Campell and Shiller 1988), price-earnings ratio (Campell and Shiller 1988; Welch and Goyal 2008), short interest rate (Campbell 1987; Ang and Bekaert 2007), term and default spreads (Campbell 1987; Fama and French 1989), and consumption-wealth ratio (Lettau and Ludvigson 2001). Besides predictors, forecasting techniques also play an important role in determining forecast accuracy. According to Mallikarjuna and Rao (2019), traditional regression techniques generally outperform others including artificial intelligence and frequency domain models in providing accurate forecasts.

In terms of stock volatility, academic researchers used to make the forecasts by traditional GARCH models using indicators based on the past behavior of stock price and volatility (Gokcan 2000; Emenogu et al. 2020). More recent studies become aware of issues such as parametric assumptions, leverage and asymmetric effects, and power transformations and long memory (e.g., Brooks 2007; Bandi and Reno 2012; Hou 2013). In this paper, we introduce GARCH models for volatility forecasting because we aim to test for the instability of the volatility process, which is primarily built upon those modeling techniques.

### Major events and stock market performance

Return and price volatility are two important indicators of market performance. Their changes can be divided into two categories: small changes and large jumps/declines (or alternatively structural changes). According to Glosten and Milgorm (1985), the former is triggered by information flows or liquidity shifts while the latter is induced by major events, including financial crises, policy changes and natural disasters. For example, Schwert (2011) shows historically high levels of stock market volatility in the months following the financial crisis in late 2008. By using data of 13 OECD countries over the period from 1972 to 2002, Ioannidis and Kontonikas (2008) find that monetary policy shifts significantly affect stock returns. Landfear et al. (2019) document abnormal negative effects on stock returns due to the U.S. landfall hurricanes.

As a public crisis, COVID-19 is inflicting unprecedented global destructive economic damage (Phan and Narayan 2020). In a recent pioneer study, Goodell (2020) highlights that COVID-19 may have wide ranging influence on financial sectors including stock markets. Empirical evidence also supports the statement. For example, based on data of 64 (advanced and emerging) countries over the period January 22, 2020 to April 12, 2020, Ashraf (2020a, b) find that stock markets negatively react to COVID-19 and this reaction varies over time depending on the stage of the outbreak. When extending data to 77 countries’ main indices, Liu et al. (2020b) reinforce that the pandemic incurs considerable negative shocks on global stock markets. Topcu and Gulal (2020) draw a similar conclusion when only focusing on emerging markets. Whether the effect of COVID-19 on stock markets is transient or permanent depends on the nature of the markets (Gil-Alana and Claudio-Quiroga 2020). Although recent literature reports that global stock markets react to the COVID-19 pandemic with negative returns, Ashraf (2020a, b) find uniform reaction across countries: the response is stronger for countries with higher national level uncertainty aversion.

With respect to volatility, Baker et al. (2020) point out that “COVID-19 has resulted in the highest stock market volatility among all recent infectious diseases including the Spanish Flu of 1918”. This is also supported by Baig et al. (2020). Sharma (2020) further shows that COVID-19 has a statistically significant effect on stock volatility, but the impact actually varies with countries involved, with the markets in higher-income countries overreacting in the beginning and bouncing back more rapidly than lower-income countries. Engelhardt et al. (2020), on the other hand, argue that the magnitude of market volatility in reaction to COVID-19 depends on trust: volatility is significantly lower in high-trust (including societal trust and trust in the government) countries.

### Structural break tests

Structural break tests for regression models (such as return prediction models) can be dated back to Chow (1960), who develops an *F*-test for a single break assuming the date for the break is known. Other tests for a single unknown break are Brown et al. (1975), Andrews (1993) and Andrews et al. (1996) for instance. More recent studies extend the prior research to allow for multiple breaks, unit root dynamics, heteroskedasticity and serial correlation (e.g., Bai and Perron 1997, 1998, 2003; Elliott and Muller 2004; Lee et al. 2021). Empirical investigation based on those recent econometric techniques includes Paye and Timmermann (2006), Rapach and Wohar (2006), and Hong et al. (2018). In this paper, we consider the methodology of Bai and Perron (1998, 2003) because it allows us to determine the confidence intervals for the timing of break occurrence as well as the coefficients around the breakpoints. For robustness, we also include Elliot and Muller (2004) which accommodate for various types of breaks like rare, large breaks as well as those with small, frequent breaks.

There are a large number of studies on tests for structural breaks in volatility. These include the most widely used one which is based on the cumulative sum (CUSUM) of squared series. Numerous researchers have developed and empirically implemented versions of the CUMSUM test (e.g., Inclan and Tiao 1994; Lee and Park 2001; Rapach and Strauss 2008; Xu 2008). They differ in how they deal with asset return features like non-normality and serial dependence. The primary issue of the existing CUSUM-based tests, as argued by Xu (2013), is that they are constructed without any explicit alternative hypotheses. This exposes the tests to criticism for having low power in practice even though they are consistent against a broad range of alternatives. This paper thus uses the methodology of Xu (2013), which specifies an alternative that allows for both smooth and abrupt changes in volatility without compromising the diagnostic ability of the CUSUM-based test. For robustness, we also consider a modified test based on the Lagrange multiplier (LM) principle.

## Data

Our datasets consist of two main U.S. stock market indices and predictor variables of concerns to investors. It should be noted that apart from traditional predictors, we also consider sentiment and technical indicators given their importance especially when crises hit the market (e.g., Wen et al. 2019). Table 1 shows the definition, data availability, and the data source for all variables under investigation.

**Table 1:** Data description

Symbol	Variable	Definition	Data availability	Data source
${R}_{t}^{S\&P 500}$	Daily Standard and Poor’s 500 (S&P 500) Stock Index Return	$log({PI}_{t}^{S\&P500}/{PI}_{t-1}^{S\&P500})$	01/01/2019–06/30/2020	Yahoo Finance
${R}_{t}^{DJIA}$	Daily Dow Jones Industrial Average (DJIA) Stock Index Return	$log({PI}_{t}^{DJIA}/{PI}_{t-1}^{DJIA})$	01/01/2019–06/30/2020	Yahoo Finance
${SI}_{t-1}^{US}$	Daily Lagged Short Interest Rate	3-Month Treasury Bill Rate	01/01/2019–06/30/2020	Federal Reserve Bank of St. Louis
${TS}_{t-1}^{US}$	Daily Lagged Term Spread	Difference between 10-Year Treasury Bond Rate and 3-Month Treasury Bill Rate	01/01/2019–06/30/2020	Federal Reserve Bank of St. Louis
${DS}_{t-1}^{US}$	Daily Lagged Default Spread	Difference between Moody’s Seasoned Baa and Aaa Corporate Bond Yields	01/01/2019–06/30/2020	Federal Reserve Bank of St. Louis
${CVIX}_{t-1}^{US}$	Daily Lagged Change in Chicago Board of Options Exchange (COBE) Volatility Index (VIX)	$log ({VIX}_{t}^{US}/{VIX}_{t-1}^{US})$	01/01/2019–06/30/2020	www.Investing.com
${CTV}_{t-1}^{S\&P 500}$	Daily Lagged Change in S&P500 Trading Volume	$log ({CTV}_{t}^{S\&P500}/{CTV}_{t-1}^{S\&P500})$	01/01/2019–06/30/2020	Yahoo Finance
${CTV}_{t-1}^{DJIA}$	Daily Lagged Change in DJIA Trading Volume	$log ({CTV}_{t}^{DJIA}/{CTV}_{t-1}^{DJIA})$	01/01/2019–06/30/2020	Yahoo Finance
${R}_{t-1}^{S\&P 500}$	Daily Lagged Standard and Poor’s 500 (S&P 500) Stock Index Return	$log ({PI}_{t-1}^{S\&P500}/{PI}_{t-2}^{S\&P500})$	01/01/2019–06/30/2020	Yahoo Finance
${R}_{t-1}^{DJIA}$	Daily Dow Jones Industrial Average (DJIA) Stock Index Return	$log ({PI}_{t-1}^{DJIA}/{PI}_{t-2}^{DJIA})$	01/01/2019–06/30/2020	Yahoo Finance

Table 1 shows primary information for both stock index returns and predictor variables. $PI=$ the stock price index adjusted for both dividends and splits; $VIX=$ volatility index. Subscripts $t$ and $t-1$ are day $t$ and $t-1$, respectively

There are several important observations to be noted. First, daily data are employed for a more precise detection of structural breaks in regression models. Therefore, traditional predictors such as the dividend yield, price-earnings ratio and the consumption-wealth ratio and those of interest to investors such as the unemployment rate are not included due to their data frequency. Second, a relatively short period January 1st, 2019 to June 30th, 2020 is introduced because a longer period might increase the power of the test but also introduce undesired noises, which would make our linkage of potential breaks with COVID-19 much harder. Third, the length of lag 1 only is considered as daily stock prices tend to rapidly incorporate publicly available information.

Table 2 illustrates the descriptive analysis of those stock index returns and predictor variables. Observations highlight that from January 1st, 2019 to June 30th, 2020 the average index returns were 0.0002 and 0.0001 for S&P 500 and DJIA, respectively. The kurtosis values of the returns (more than 16.0000) indicate that large jumps and extreme movements were prevalent in both markets. This may attribute to the four consecutive triggers of the key market-wide circuit breaker on March 9th, 12th, 16th and 18th, 2020. Both the short interest rate and the term spread (also known as indictors for future economic activities) fell below zero and the change in VIX (also referred to as ‘the fear gauge’) dropped to as low as − 0.1156 (compared to its highest level 0.1701) during the same period, indicating potential economic contractions.

**Table 2:** Summary Statistics: 01/01/2019–06/30/2020

Variable	Mean	STD	Min	Max	Skewness	Kurtosis	Number of Obs.
${R}^{S\&P 500}$	0.0002	0.0078	− 0.0554	0.0389	− 1.0167	16.6603	377
${R}^{DJIA}$	0.0001	0.0083	− 0.0601	0.0467	− 1.0053	17.5810	377
${SI}^{US}$	1.6079	0.8402	− 0.0460	2.4730	− 0.8568	2.3110	377
${TS}^{US}$	0.1596	0.2966	− 0.5240	1.1630	0.1647	2.8855	377
${DS}^{US}$	1.0644	0.2477	0.7900	1.9900	1.6558	5.5371	377
${CVIX}^{US}$	0.0002	0.0383	− 0.1156	0.1701	1.2662	6.6640	377
${CTV}^{S\&P 500}$	0.0004	0.0780	− 0.3730	0.2742	− 0.4012	7.2318	377
${CTV}^{DJIA}$	0.0004	0.1010	− 0.4126	0.3744	− 0.1001	5.9482	377

Table 2 reports the mean, standard deviation (STD), minimum (Min), maximum (Max), skewness, kurtosis, and the number of observations (Num of Obs) for both stock index returns and predictor variables over the period January 1st, 2019 to June 30th, 2020

## COVID-19 and instability of stock return predictability

We begin by investigating the role of COVID-19 on the instability of the U.S. stock return predictability. First, we focus on establishing reasonable regression models to predict future returns. Second, we test for the presence, location and the significance of structural breaks in the return prediction models. Next, we investigate whether the derived breaks can be related to COVID-19.

### Stock return predictions

As shown in Table 1, predictor variables available to use for predicting stock index returns ${R}_{t}^{S\&P 500}$ and ${R}_{t}^{DJIA}$ include ${SI}_{t-1}^{US}$, ${TS}_{t-1}^{US}$, ${DS}_{t-1}^{US}$, ${CVIX}_{t-1}^{US}$, ${CTV}_{t-1}^{S\&P 500}$, ${CTV}_{t-1}^{DJIA}$, ${R}_{t-1}^{S\&P 500}$ and ${R}_{t-1}^{DJIA}$. Before the formal model setup, we test the correlations between all the variables and present the results of the correlation matrix in Table 3. It is apparent that ${DS}_{t-1}^{US}$, ${CVIX}_{t-1}^{US}$ and ${R}_{t-1}$ have a strong relationship with both index returns.

**Table 3:** Correlation Matrix: 01/01/2019–06/30/2020

Variable	${R}_{t}^{S\&P 500}$	${SI}_{t-1}^{US}$	${TS}_{t-1}^{US}$	${DS}_{t-1}^{US}$	${CVIX}_{t-1}^{US}$	${CTV}_{t-1}^{S\&P 500}$	${R}_{t-1}^{S\&P 500}$
*Panel A. S&P 500*							
${R}_{t}^{S\&P 500}$	1.000	0.0018 (0.9725)	− 0.0134 (0.7962)	0.1100 (0.0330**)	0.1641 (0.0014***)	0.0001 (0.9980)	− 0.3397 (0.0000***)
${SI}_{t-1}^{US}$		1.0000	− 0.6905 (0.0000***)	− 0.5926 (0.0000***)	− 0.0021 (0.9677)	0.0103 (0.8427)	0.0136 (0.7930)
${TS}_{t-1}^{US}$			1.0000	0.6319 (0.0000***)	− 0.0800 (0.1216)	− 0.0206 (0.6907)	0.0761 (0.1408)
${DS}_{t-1}^{US}$				1.0000	− 0.1201 (0.0198**)	− 0.0337 (0.5146)	0.1316 (0.0106**)
${CVIX}_{t-1}^{US}$			−		1.0000	0.2235 (0.0000***)	− 0.7099 (0.0000***)
${CTV}_{t-1}^{S\&P 500}$						1.0000	− 0.1041 (0.0437**)
${R}_{t-1}^{S\&P 500}$							1.0000

Variable	${R}_{t}^{DJIA}$	${SI}_{t-1}^{US}$	${TS}_{t-1}^{US}$	${DS}_{t-1}^{US}$	${CVIX}_{t-1}^{US}$	${CTV}_{t-1}^{DJIA}$	${R}_{t-1}^{DJIA}$
*Panel B. DJIA*							
${R}_{t}^{DJIA}$	1.0000	0.0049 (0.9248)	− 0.0170 (0.7428)	0.1062 (0.0395**)	0.1593 (0.0019***)	0.0181 (0.7271)	− 0.3123 (0.0000***)
${SI}_{t-1}^{US}$		1.0000	− 0.6905 (0.0000***)	− 0.5926 (0.0000***)	− 0.0021 (0.9677)	0.0085 (0.8691)	0.0144 (0.7802)
${TS}_{t-1}^{US}$			1.0000	0.6319 (0.0000***)	− 0.0800 (0.1216)	− 0.0206 (0.6907)	0.0718 (0.1605)
${DS}_{t-1}^{US}$				1.0000	− 0.1201 (0.0198**)	− 0.0395 (0.4454)	0.1291 (0.0123**)
${CVIX}_{t-1}^{US}$					1.0000	0.2608 (0.0000***)	− 0.6809 (0.0000***)
${CTV}_{t-1}^{DJIA}$						1.0000	− 0.1331 (0.0098***)
${R}_{t-1}^{DJIA}$							1.0000

Table 3 reports the pairwise Pearson correlations between all variables over the period from January 1st, 2019 to June 30th, 2020. *P* values are provided in the parentheses

** and ***indicate significance at the 5% and 1% levels, respectively

In order to establish reasonable regression models for stock return predictions, we employ the widely-adopted stepwise methodology embedded with Bayesian Information Criterion (BIC) to select predictor variables (e.g., Hsu et al. 2010; Hong et al. 2018). It should be noted that we aim to establish regression models that can be used to predict stock returns rather than choose the best statistical models for predictions. The procedure begins with a constant model. At each step the BIC is computed to compare models with and without a predictor variable and the variable is then added or removed from the model accordingly. This procedure terminates when no variable can be introduced and eliminated. The final model takes the form given by:

1 $$R_{t} = \beta z_{t - 1} + \varepsilon_{t} ,$$

where $R=$ the log of stock index returns as described in Table 1; $z=$ the vector of the constant and predictor variables selected by the stepwise regression. $\varepsilon =$ the disturbance term with mean zero and variance ${\sigma }^{2}$. Subscripts $t$ and $t-1$ are day $t$ and $t-1$, respectively.

Table 4 provides estimation results for predictive regression models, including the estimated coefficients of the selected predictor variables and their standard errors, adjusted ${R}^{2}$ and the root mean square errors (RMSEs). To save space, we only report estimates of the final models.

**Table 4:** Estimation Results of Stepwise Predictive Regression Models and Robustness of Parameter Estimates: 01/01/2019–06/30/2020

	Least-Square Regression Statistics			Outlier Robust Statistics^a^			Heteroskedasticity and Serial Correlation Statistics^b^		
	Estimate	Adjusted ${R}^{2}$	RMSE	Estimate	Adjusted ${R}^{2}$	RMSE	Estimate	Adjusted ${R}^{2}$	RMSE
*Model I: *${R}_{t}^{S\&P 500}={\beta }_{0}^{S\&P 500}+{\beta }_{1}^{S\&P 500}{DS}_{t-1}^{US}+{\beta }_{2}^{S\&P 500}{R}_{t-1}^{S\&P 500}+{\varepsilon }_{t}$									
${\widehat{\beta }}_{0}^{S\&P 500}$	− 0.0050 (0.0017***)	0.1352	0.0073	− 0.0035 (0.0008***)	0.1905	0.0037	− 0.0050 (0.0021**)	0.1352	0.0073
${\widehat{\beta }}_{1}^{S\&P 500}$	0.0050 (0.0015***)			0.0043 (0.0008***)			0.0050 (0.0020**)		
${\widehat{\beta }}_{2}^{S\&P 500}$	− 0.3608 (0.0485***)			− 0.1384 (0.0254***)			− 0.3608 (0.1054***)		
*Model II:*${R}_{t}^{DJIA}={\beta }_{0}^{DJIA}+{\beta }_{1}^{DJIA}{{DS}_{t-1}^{US}+\beta }_{2}^{DJIA}{R}_{t-1}^{DJIA}+{\varepsilon }_{t}$									
${\widehat{\beta }}_{0}^{DJIA}$	− 0.0052 (0.0018***)	0.1146	0.0079	− 0.0035 (0.0009***)	0.1322	0.0038	− 0.0052 (0.0021**)	0.1146	0.0079
${\widehat{\beta }}_{1}^{DJIA}$	0.0050 (0.0017***)			0.0042 (0.0008***)			0.0050 (0.0022**)		
${\widehat{\beta }}_{2}^{DJIA}$	− 0.3316 (0.0490***)			− 0.2354 (0.0248***)			− 0.3316 (0.1022***)		

Table 4 reports both estimation and robust estimation results of the final predictive regression models determined by the BIC over the period January 1st, 2019 to June 30th, 2020

^a^Outlier robust statistics are obtained using iteratively reweighted lease squares with a bi-square weighting function

^b^The Newey-West statistics are used as the heteroskedasticity and serial correlation robust statistics, where the lag calculated as $4*{(\frac{N}{100})}^{2/9}$ is introduced to correct for serial correlation. Standard errors are provided in the parentheses

** and *** indicate significance at the 5% and 1% levels, respectively

The table highlights the inclusion of ${DS}_{t-1}^{US}$ and ${R}_{t-1}^{S\&P 500}$ in the S&P 500 return model and ${DS}_{t-1}^{US}$ and ${R}_{t-1}^{DJIA}$ in the DJIA return model. The estimated coefficients for both models are statistically significant at the 1% level with hypothesized signs. For example, the negative coefficients of the lagged return series are consistent with the price pressure hypothesis that heavy trading of the U.S. index component stocks tends to produce price pressure or excess volatility (Vijh 1994). The adjusted ${R}^{2}$ values at a more than 10% level is non-negligible and can have important implications for asset-allocation decisions as the predictable component of stock returns is usually relatively small (Kandel and Stambaugh 1996). Moreover, these regressions have also reached a local minimum of RMSE. Other predictor variables listed in Table 1 are not included in the final models due to their failure to meet the selection criteria of the stepwise regression. It should also be noted that ${CVIX}_{t-1}^{US}$ are strongly correlated with ${R}_{t-1}^{S\&P 500}$ and ${R}_{t-1}^{DJIA}$ as shown in Table 3 such that only ${R}_{t-1}^{S\&P 500}$ or ${R}_{t-1}^{DJIA}$ enters the final models based on the information criteria.

The results reinforce those in prior studies that indicate an important role of economic fundamentals in explaining the U.S. stock returns (e.g., Rapach and Wohar 2006; Chang et al. 2019). They also highlight strong autocorrelations in the daily U.S. stock returns, consistent with the literature of short-term stock return behavior (e.g., Avramov et al. 2006; Bogousslavsky 2016).

Being aware of general data problems, we further test to what extent our ordinary least square (OLS) estimates might be affected and also present the results in Table 4. We evaluate the robustness of our results by testing whether parameter estimates change substantially after accounting for the effects of both outliers and heteroskedasticity and serial correlation. The table highlights the robustness of the performance of all predictive regression models to potential data issues. The estimated coefficients (i.e.,${\widehat{\beta }}_{0}^{S\&P 500}$,${\widehat{\beta }}_{1}^{S\&P 500}$,${\widehat{\beta }}_{2}^{S\&P 500}$,${\widehat{\beta }}_{0}^{DJIA}$, ${\widehat{\beta }}_{1}^{DJIA}$ and ${\widehat{\beta }}_{2}^{DJIA}$) remain significant at the same statistical level and the RMSE for each model becomes much smaller, suggesting that our OLS estimates barely suffer from the outlier effect. With respect to the heteroskedasticity and serial correlation, both S&P 500 and DJIA return models are more or less affected: for instance, the standard error is 0.0020 (0.0022) for ${\widehat{\beta }}_{1}^{S\&P 500}$ (${\widehat{\beta }}_{1}^{DJIA}$), leading to a decrease in the significance of the coefficient from 1% to 5%. However, the models remain competitive in forecasting as the coefficients are still important at the conventional level.

Overall, the results suggest that the predictive regression models derived is reasonable for predicting the U.S. stock returns, with non-significant changes in parameter estimates after accounting for potential data problems. Next section turns to whether structural breaks exist and how they may affect model parameters.

### Tests for breaks and their significance

As mentioned previously, we introduce Bai and Perron (1998, 2003) and Elliot and Mullier (2004) to test for unknown structural breaks. The former enables estimation of breakpoints and statistical analysis of the resulting estimators whereas the latter displays excellent properties in the presence of persistence (given the non-stationarity of the default spread in the final regression models). In our implementation, we allow all coefficients to change at each break since there is no strong reason to believe that any coefficient involved should be immune from shifts.

Table 5 reports the results of various tests for structural breaks which allow for serial correlation, different variances of the errors and trending regressors as well as those for the *true* number of breaks. The selection of trimming percentages ($\pi$) is vital to break test results since a small $\pi$ can cause substantial size distortions while a large $\pi$ can sharply reduce the combinations of breakpoints allowed. Usually, there are five options for $\pi s$: 5%, 10%, 15%, 20%, 25% (Bai and Perron 1998). We set the trimming percentage,$\pi$, to 20% and 25%, allowing for 3 breaks ($m=3$) and 2 breaks ($m=2$), respectively given the relatively short sample period under studied. These correspond to a minimum window of approximately 94 and 126 days between breaks for our datasets starting from January 1st, 2019.

**Table 5:** Bai and Perron (1998, 2003) and Elliot and Mueller (2004)’ Test Statistics for Breaks and Model Selection: 01/01/2019–06/30/2020

*SupF* (1)	*SupF* (2)	*SupF* (3)	*UD*max^a^	*WD*max	*SupF*_*T*_(1|0)	*SupF*_*T*_(2|1)	*SupF*_*T*_(3|2)	$\widehat{J}$*-*Stat	BIC	LWZ	SM
Panel A: $\pi =20\mathrm{\%},m=3\mathrm{ breaks}$											
Model I: ${R}_{t}^{S\&P 500}={\beta }_{0}^{S\&P 500}+{\beta }_{1}^{S\&P 500}{DS}_{t-1}^{US}+{\beta }_{2}^{S\&P 500}{R}_{t-1}^{S\&P 500}+{\varepsilon }_{t}$											
8.6434^**^	5.5298	4.2090	8.6434**	8.6434**	7.4733*	3.4279	0.0000	− 16.9766**	1	0	1
Model II: ${R}_{t}^{DJIA}={\beta }_{0}^{DJIA}+{\beta }_{1}^{DJIA}{{DS}_{t-1}^{US}+\beta }_{2}^{DJIA}{R}_{t-1}^{DJIA}+{\varepsilon }_{t}$											
7.4514^*^	5.1430	3.6911	7.4514*	7.4514*	6.9766*	5.0930	0.2501	− 13.8949*	1	0	1
Panel B:$\pi =25\mathrm{\%},m=2\mathrm{ breaks}$											
Model I: ${R}_{t}^{S\&P 500}={\beta }_{0}^{S\&P 500}+{\beta }_{1}^{S\&P 500}{DS}_{t-1}^{US}+{\beta }_{2}^{S\&P 500}{R}_{t-1}^{S\&P 500}+{\varepsilon }_{t}$											
7.9936^*^	5.1009	/	7.9936*	7.9936*	7.5632*	3.4504	–	− 14.1311*	1	0	1
Model II: ${R}_{t}^{DJIA}={\beta }_{0}^{DJIA}+{\beta }_{1}^{DJIA}{{DS}_{t-1}^{US}+\beta }_{2}^{DJIA}{R}_{t-1}^{DJIA}+{\varepsilon }_{t}$											
7.3832*	5.1663	/	7.3832*	7.3832*	6.9766*	5.1307	–	− 13.8012*	1	0	1

Table 5 reports the results of various test for structural breaks in both S&P 500 and DJIA return models and those for the number of *true* breaks over the period January 1st, 2019 to June 30th, 2020. The *Sup*F statistics are used to test the hypothesis of no structural break against the one-sided (upper-tail) alternative of *m = k* (*k* is a predefined number). The double maximum statistics and the *J*-test statistic are used to test hypothesis of no structural break against the one-sided (upper-tail) alternative of an unknown number of breaks. The ${Sup\mathrm{F}}_{\mathrm{T}}(l+1|l)$ statistics have the null hypothesis of *l* breaks against the one-sided (upper-tail) alternative of *l+1* breaks. The Bayesian information criterion (BIC) (Yao 1988), a modified Schwarz criterion (LWZ) (Liu et al. 1997) and the sequential method (SM) (Bai and Perron 1998) are used to test the number of *true* breaks. The ${Sup\mathrm{F}}_{\mathrm{T}}(l+1|l)$ test in the sequential context is different from the sequential method because the first *l* breaks are not necessarily the global minimizers of the sum of squared residuals. The statistics allow for the possibility of heteroscedasticity and serial correlation in the errors. The heteroscedasticity and autocorrelation consistent covariance matrix is constructed following Andrews (1991) and Andrews and Monahan (1992) using a quadratic kernel with automatic bandwidth selection based on a first order Vector Auto-regression (VAR (1)) approximation. The autocorrelation from the residuals is removed also using a VAR (1)

* and **indicate significance at the 10% and 5% levels, respectively

As expected, the statistics of the $SupF$(1) test, double maximum tests, ${SupF}_{t}\left(1|0\right)$ test and the $\widehat{J}$-test are all significant for both $\pi =20\%$ and $\pi =25\%$, suggesting the presence of structural breaks (highly likely a break). Together with the evidence of the BIC and the SM for the number of *true* breaks, we conclude with one-break prediction models for both S&P 500 and DJIA returns. These findings reinforces those in prior studies that predictive regression models are characterized by structural instability (e.g., Rapach and Wohar 2006; Paye and Timmermann 2006; Hong et al. 2018).

Although the hypothesis tests above suggest statistical significance of instability, they alone cannot reveal the economic significance of the break. To this end, we re-estimate both S&P 500 and DJIA return models in different regimes partitioned by the derived break above. Table 6 reports the estimated coefficients and their standard errors, adjusted ${R}^{2}$, and also the estimated breakpoint as well as its 90% confidence interval.

**Table 6:** Estimation Results of Multiple Regime Regression Models: 01/01/2019–06/30/2020

	Regime 1			Regime 2		
	(1)	(2)	(3)	(4)	(5)	(6)
	Estimate	Adjusted ${R}^{2}$	Breakpoint	Estimate	Adjusted ${R}^{2}$	Breakpoint
Panel A:$\pi =0.20,m=3\mathrm{ breaks}$						
Model I: ${R}_{t}^{S\&P 500}={\beta }_{0}^{S\&P 500}+{\beta }_{1}^{S\&P 500}{DS}_{t-1}^{US}+{\beta }_{2}^{S\&P 500}{R}_{t-1}^{S\&P 500}+{\varepsilon }_{t}$						
${ \widehat{\beta }}_{0}^{S\&P 500}$	− 0.0017 (0.0014)	0.0065	02/20/2020 [01/02/2020–02/25/2020]	− 0.0232 (0.0076***)	0.2488	–
${ \widehat{\beta }}_{1}^{S\&P 500}$	0.0023 (0.0015)			0.0166 (0.0054***)		
${\widehat{\beta }}_{2}^{S\&P 500}$	− 0.0973 (0.0585*)			− 0.4766 (0.1108***)		
Model II: ${R}_{t}^{DJIA}={\beta }_{0}^{DJIA}+{\beta }_{1}^{DJIA}{{DS}_{t-1}^{US}+\beta }_{2}^{DJIA}{R}_{t-1}^{DJIA}+{\varepsilon }_{t}$						
${\widehat{\beta }}_{0}^{DJIA}$	− 0.0023 (0.0015)	0.0124	02/21/2020 [12/20/2019–02/25/2020]	− 0.0244 (0.0085***)	0.2075	–
${ \widehat{\beta }}_{1}^{DJIA}$	0.0027 (0.0015*)			0.0173 (0.0060***)		
${ \widehat{\beta }}_{2}^{DJIA}$	− 0.1170 (0.0635*)			− 0.4333 (0.1158***)		
Panel B:$\pi =0.25,m=2\mathrm{ breaks}$						
Model I: ${R}_{t}^{S\&P 500}={\beta }_{0}^{S\&P 500}+{\beta }_{1}^{S\&P 500}{DS}_{t-1}^{US}+{\beta }_{2}^{S\&P 500}{R}_{t-1}^{S\&P 500}+{\varepsilon }_{t}$						
${ \widehat{\beta }}_{0}^{S\&P 500}$	− 0.0017 (0.0015)	0.0063	02/14/2020 [12/17/2019–02/20/2020]	− 0.0208 (0.0069***)	0.2367	–
${ \widehat{\beta }}_{1}^{S\&P 500}$	0.0023 (0.0015)			0.0150 (0.0047***)		
${ \widehat{\beta }}_{2}^{S\&P 500}$	− 0.0963 (0.0586)			− 0.4688 (0.1117***)		
Model II:${R}_{t}^{DJIA}={\beta }_{0}^{DJIA}+{\beta }_{1}^{DJIA}{{DS}_{t-1}^{US}+\beta }_{2}^{DJIA}{R}_{t-1}^{DJIA}+{\varepsilon }_{t}$						
${ \widehat{\beta }}_{0}^{DJIA}$	− 0.0021 (0.0016)	0.0112	02/14/2020 [12/04/2019–02/19/2020]	− 0.0214 (0.0076***)	0.1963	–
${\widehat{\beta }}_{1}^{DJIA}$	0.0026 (0.0016)			0.0153 (0.0054***)		
${ \widehat{\beta }}_{2}^{DJIA}$	− 0.1157 (0.0638*)			− 0.4235 (0.1149***)		

Table 6 reports least-square estimation results of both S&P 500 and DJIA return models in different regimes based on the derived break over the period January 1st, 2019 to June 30th, 2020. Standard errors in parentheses are Newey-West standard errors adjusted for heteroskesticity and serial correlation. 90% confidence interval for the breakpoint is reported in the bracket

* and ***indicate significance at the 10% and 1% levels, respectively

The regressions reveal several interesting results. Most notably, the predictive regression coefficients as well as the adjusted ${R}^{2}$ values of both S&P 500 and DJIA return models change substantially following the break. For instance, in the case of $\pi =0.20$, the estimated coefficient of ${DS}_{t-1}^{US}$ for Model I was 0.0023 in regime 1 (January 1st, 2019 to February 19th, 2020). It increased to 0.0166 with significance at the 1% level when one moves to regime 2 (February 20^th^, 2020 to June 30th, 2020). The adjusted ${R}^{2}$ value was 0.0065 before the break and increased substantially to 0.2488 afterwards. While some of this variation can be clearly attributed to sampling variation due to sometimes large standard errors, this does not conceal the fact that the derived break in the regression models tends to be sufficiently large that it is of substantial economic interest. It should also be noted that parameter specification has little impact on the break location: the breakpoint (02/20/2020 or 02/21/2020) when $\pi =0.20$ is quite close to (02/14/2020) when $\pi =0.25$. The difference is actually induced by a minimum window allowed between each break.

### Nature of the break

Questions may naturally arise about what the primary cause for the derived break is. We examine whether the timing of the statistical break is consistent with the COVID outbreak. According to the COVID-19 data repository by the center for systems science and engineering (CSSE) at Johns Hopkins university, the first case of COVID-19 was confirmed in the U.S on January 22nd, 2020. The breakpoint 02/20/2020 (02/21/2020) when $\pi =0.20$ and that 02/14/2020 when $\pi =0.25$ for the S&P 500 (DJIA) return model are evidently late than the start of the pandemic though their 90 percent confidence intervals are not precisely estimated as they cover a broad range of dates.

**Fig 1.**
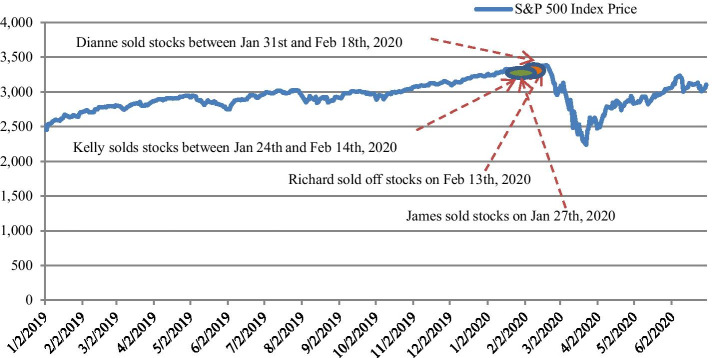
Stock Selling by the Senate Committee Members and the performance of the S&P 500: 01/01/2019–06/30/2020. *Notes*: Figure 1 plots the timing of the stock selling by the senate committee members over the period January 1st, 2019 to June 30th, 2020

A close look at those breakpoints derived reveals interesting facts that they seem to approach the end of the stock selling by senate committee members in the U.S after COVID-19 broke out. According to Fox News, 4 senators sold off stocks worth millions of dollars in the days before the coronavirus outbreak crashed the market. Specifically, Richard Burr, chairman of the Senate Intelligence Committee, conducted more than 30 transactions to dump between $628,000 and $1.72 million on February 13th, 2020. Dianne Feinstein, ranking member of the Senate Judiciary Committee, and her husband sold between $1.5 million and $6 million between January 31st, 2020 and February 18th, 2020. Kelly Loeffler and her husband, Jeffrey Sprecher, Chairman of the New York Stock Exchange, sold stocks between January 24th, 2020 and February 14th, 2020, worth a total between $1.2 million and $3.1million. James Inhofe sold as much as $400,000 on January 27th, 2020. Figure 1 further visualizes the timing of the stock selling by the senate committee members. It is apparent that the market plunged immediately after those senators dumped their stocks. This further indicates that information asymmetry existed between government bureaucrats and the public. Bureaucrats have information advantage, enabling them to successfully reduce the likelihood of huge losses during the COVID-19 crisis.

Overall, we find that stock return predictability was subject to a structural break which can be ascribed to COVID-19 during the period under investigation. Moreover, the predictability significantly increased after the outbreak of the pandemic crisis. According to Cujean and Hasler (2017), as economic conditions deteriorated, difference in investors’ learning speed increased. Investors’ opinions eventually polarized, causing returns to react to past information.

The findings have vast implications for academic researchers, investors and policy makers. First, COVID-19 is an important cause of market inefficiency, implying significant return predictability and existence of profitable opportunities for traders and speculators. The selling-offs by insiders provides clues to market timing. Second, profitable opportunities will benefit those who have plenty of liquidity at hand. Third, Fed policy of pumping liquidity into the financial system may have stimulated profitability seeking in the stock market, which may enlarge income and wealth inequality.

## COVID-19 and instability of price volatility

In this section, we turn to studying the characteristics of price volatility in the COVID-19 context. As with return predictability, first, we attempt to establish reasonable models for modeling price volatility. Second, we focus on testing for the presence, location and the significance of structural breaks in volatility models. Third, we examine the linkage between the derived breaks and COVID-19.

### Price volatility modeling

**Fig 2.**
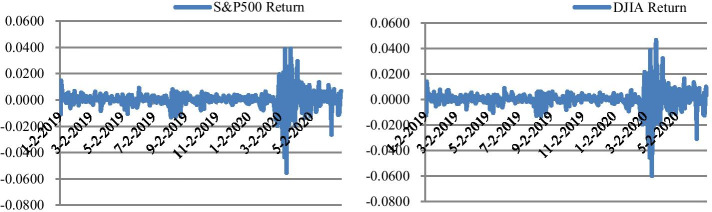
Time Series Plots: 01/01/2019–06/30/2020*. Notes*: Figure 2 plots the time series of both S&P 500 and DJIA for the period January 1st, 2019 to June 30th, 2020

Before the formal model set up, we investigate stock return behavior for the period studied. Figure 2 plots return series of both S&P 500 and DJIA. It is apparent that large or small changes in prices tend to cluster together, resulting in the persistence of these magnitudes of price changes. Supportive evidence is from the tests for ARCH effect (lag 15) in Table 7: statistics are significant at the 1% level for both return series, indicating potential high-level ARCH effect, i.e., GARCH effect.

**Table 7:** Tests for ARCH Effect: 01/01/2019–06/30/2020

Return	ARCH (15) Statistics	Return	ARCH (15) Statistics
${R}_{t}^{S\&P 500}$	164.1490***	${R}_{t}^{DJIA}$	158.4570***

Table 7 reports statistics of ARCH effect for both S&P 500 and DJIA over the period January 1st, 2019 to June 30th, 2020. ARCH lags are determined by BIC

***indicates significance at the 1% level

The GARCH model involved is then given by:

2 $$\begin{aligned} R_{t} & = \sigma_{t} \varepsilon_{t} , \\ \sigma_{t}^{2} & = \alpha_{0} + \alpha_{1} \varepsilon_{t - 1}^{2} + \cdots + \alpha_{q} \varepsilon_{t - q}^{2} + \beta_{0} + \beta_{1} \sigma_{t - 1}^{2} + \cdots + \beta_{p} \sigma_{t - p}^{2} , \\ \end{aligned}$$

where $\upsigma =$ non-stationary unconditional variance; $\varepsilon =$ potential conditional heteroscedasticity. $R$ is the same as in Eq. (1). ${\upsigma }_{t}$ is a deterministic function of *t* and ${\varepsilon }_{t}$ satisfies $\mathrm{E}\left({\varepsilon }_{t}\right)=0$ and $\mathrm{E}\left({\varepsilon }_{t}^{2}\right)=1$. To determine the orders of the GARCH models in Eq. (2), we consider several potential possibilities (Mcmillan and Speight 2004) and select the orders by BIC. Table 8 shows that the BIC values increase as orders of the models become larger, thus supporting GARCH (1,1) as the optimal model for describing the volatility process. This is consistent with Ashely and Patterson (2010) who find that GARCH (1,1) is in general adequate for modeling daily price volatility.

**Table 8:** Tests for GARCH effect: 01/01/2019–06/30/2020

	${\alpha }_{0}$	${\alpha }_{1}$	${\alpha }_{2}$	${\beta }_{1}$	${\beta }_{2}$	*L*	BIC
*Panel A: S&P 5000*							
GARCH (1,1)	0.0000 (0.0000***)	0.2642 (0.0038***)	–	0.7356 (0.0018***)	–	1503.0000	− 5.9455
GARCH (2,1)	0.0000 (0.0000***)	0.1810 (0.0104**)	0.1606 (0.0093***)	0.6582 (0.0011***)	–	1504.5000	− 5.8902
GARCH (2,2)	0.0000 (0.0000***)	0.1803 (0.0085***)	0.3224 (0.0040***)	0.0833 (0.0011***)	0.4138 (0.0011***)	1505.2000	− 5.8865
*Panel B: DJIA*							
GARCH (1,1)	0.0000 (0.0000***)	0.2477 (0.0041***)	–	0.7521 (0.0021***)	–	1486.7000	− 5.9201
GARCH (2,1)	0.0000 (0.0000***)	0.1409 (0.0051***)	0.1709 (0.0049***)	0.6835 (0.0088 ***)	–	1488.5000	− 5.8764
GARCH (2,2)	0.0000 (0.0000***)	0.1383 (0.0016***)	0.3316 (0.0195**)	0.0949 (0.0073***)	0.4350 (0.0030***)	1489.4000	− 5.8658

Table 8 displays results of tests for the GARCH effect for both S&P 500 and DJIA over the period January 1st, 2019 to June 30th, 2020. L and BIC are presented in log values

*P* values are provided in the parentheses

** and *** indicate significance at the 5% and 1% levels, respectively

### Tests for breaks and their significance

As mentioned previously, we introduce Xu (2013) to test for unknown structural breaks in price volatility, which accommodates the stylized facts that previous studies always lack explicit alternative hypotheses, potentially leading to lower power of tests in practice. Table 9 reports the results for both modified CUSUM and LM tests ($\widehat{Q}$ and $\widehat{L}$ statistics, respectively) for the presence and the number of breaks.

**Table 9:** Xu (2013)’s Test Statistics for Breaks: 01/01/2019–06/30/2020

Model	$\widehat{Q}$ Statistic	$\widehat{L}$ Statistic	Breakpoint	No of Obs.
*Panel A: S&P 500*				
GARCH (1,1)	1.3766**	9.8543**	2020.02.21	377
*Panel B: DJIA*				
GARCH (1,1)	1.3699**	9.7405**	2020.02.21	377

Table 9 reports the results of modified CUSUM and LM tests for structural breaks in volatilites of both S&P 500 and DJIA over the period January 1st, 2019 to June 30th, 2020. The modified CUSUM test allows for multiple structural breaks while the modified LM test only allows for a single break. The bandwidth is selected by cross validation. $\widehat{Q}$ is the modified CUSUM test statistic and $\widehat{L}$ is the modified LM test statistic

**indicates significance at the 5% level

The table highlights that structural breaks are present in volatilities of S&P 500 and DJIA: both modified CUSUM and LM tests reject the constant variance hypothesis at 5% level when cross validated bandwidths are used. Further investigation indicates a single break with its location around February 21st, 2020, which is similar to the derived break in return predictability.

**Fig 3.**
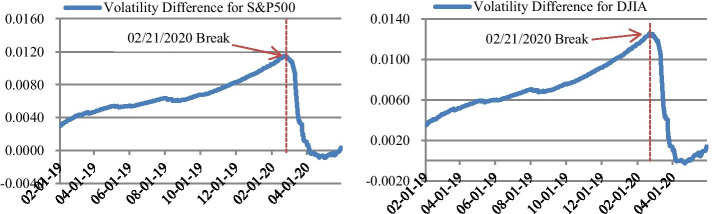
Difference of Volatility between Subsamples: 02/01/2019–05/31/2020. *Notes*: Figure 3 plots the difference of the realized volatility between two subsamples partitioned at a given date

Figure 3 plots the difference of the realized volatility between two subsamples partitioned (i.e. volatility of subsample 1 minus subsample 2) at a given date. Assume that the minimum length of time to compute volatility is one month. The sample period now becomes February 1st, 2019 to May 31st, 2020. It is apparent that the difference of the volatility between subsamples arrives at its maximum (0.0115 for S&P 500 and 0.0126 for DJIA) when the derived break above is used for sample partition. In other words, volatility significantly increased during the period after the break.

### Nature of the break

Similar to the case of return predictability, we argue that the timing of the statistical break that took place on February 21st, 2020 is consistent with the COVID-19 outbreak. More specifically, the break occurrence followed closely to the stock selling-offs by the senate committee members in the U.S before COVID-19 crashed the market.

As with Geanakoplos (2003) who argues that bad news tends to cause panic among investors such that a crisis is usually accompanied by high volatility, COVID-19 creates better investment opportunities for investors with volatility timing ability (especially those who have plenty of liquidity at hand) than the public. This however may enlarge income and wealth inequality.

## Conclusion

In this paper we examine the association between COVID-19 and the instability of the U.S. stock market performance (i.e., return predictability and price volatility). Using daily data from January 1st, 2019 to June 30th, 2020 and methodologies developed by Bai and Perron (1998, 2003), Elliot and Mullier (2004) and Xu (2013), we find that return predictability and price volatility of both S&P 500 and DJIA underwent a single structural break. The break can be related to COVID-19 or more specifically the stock selling-offs by the U.S. senate committee members before COVID-19 crashed the market. Moreover, both return predictability and price volatility increased significantly after the derived break.

Important implications are provided as follows. On one hand, crises may be associated with opportunities. COVID-19 is an important cause for market inefficiency, creating profitable opportunities for traders and speculators. Rational investors seeking to maximize returns may need to pay close attention to insider trading before taking any decisions in the stock market. On the other hand, crises may also induce income and wealth inequality as market participants with plenty of liquidity at hand can seek for profitability in the stock market.

Future research avenues might consider testing whether the structural shift is transient or permanent given its important policy implications. Furthermore, extending the analysis to more countries and comparing their similarities and differences would offer more insightful outcomes.

## Data Availability

Data available from the authors upon request.
